# Socio-demographic and environmental factors associated with diarrhoeal disease among children under five in India

**DOI:** 10.1186/s12889-020-09981-y

**Published:** 2020-12-07

**Authors:** Pintu Paul

**Affiliations:** Centre for the Study of Regional Development, School of Social Sciences, Jawaharlal Nehru University, New Delhi, 110067 India

**Keywords:** Diarrhoea, Socio-demographic factors, Environmental condition, Children under five, India

## Abstract

**Background:**

Globally, diarrhoea is the second leading cause of death in children under five and a major public health problem. Despite several health care initiatives taken by the government, a large proportion of children still experience diarrhoeal diseases which cause high childhood death in India. This study aims to examine the socio-demographic and environmental factors associated with diarrhoea in children under five in India.

**Methods:**

A cross-sectional study was designed using secondary data from the recent round of the National Family Health Survey (NFHS-4), conducted in 2015–16. A total of 247,743 living children below 5 years of age were included in the analysis. Bivariate and multivariate logistic regression models were carried out to assess the factors associated with childhood diarrhoeal disease.

**Results:**

In India, about 9% of under-five children experience diarrhoeal disease in the past 2 weeks preceding the survey. Children living in rural areas (Adjusted odds ratio [aOR]: 1.05; 95% CI: 1.01, 1.09), children belonged to scheduled tribe (aOR: 0.83; 95% CI: 0.79, 0.89) and other castes (aOR: 0.92; 95% CI: 0.88, 0.97), Muslim children (aOR: 1.18; 95% CI: 1.13, 1.24), and children resided in the central (aOR: 1.61; 95% CI: 1.52, 1.70) and west (aOR: 1.08; 95% CI: 1.01, 1.15) regions were significantly associated with higher likelihood of diarrhoea in the past 2 weeks. Concerning environmental factors, child stool disposal (aOR: 1.06; 95% CI: 0.98, 1.09), floor materials (aOR: 1.08; 95% CI: 1.03, 1.12) and roof materials (aOR: 1.08; 95% CI: 1.04, 1.13) of the household were found to be significant predictors of childhood diarrhoea occurrence.

**Conclusions:**

Diarrhoeal disease is common among children who lived in rural areas, scheduled castes, Muslims, and children from poor families. Regarding environmental factors, stool disposal practices in the household, dirt floor, and thatch roof materials of the household unit are risk factors for diarrhoeal disease. Targeted approach should be initiated to mitigate the problem of the poor health status of children by providing adequate health care. The policy-makers and stakeholders should address adverse environmental conditions by the provision of latrine and improved housing facilities.

## Introduction

Childhood diarrhoea is a major public health problem in low- and middle-income countries, leading to high mortality in children under five. According to the World Health Organization (WHO), diarrhoeal disease is defined as the passage of three or more loose or liquid stools per day [1]. This preventable disease is the second leading cause of death in children under five. Each year, nearly 1.7 billion cases of childhood diarrhoeal diseases have been reported, killed around 525,000 under-five children, accounting for 8% of all deaths worldwide [1]. Most of the deaths from diarrhoea occur in children below 2 years of age. While the decrease in the episodes of childhood diarrhoea is evident globally over the past two decades, the prevalence of the disease remains at an alarming rate in many resource-poor settings where infants and children are still at risk of death and other complications such as malnutrition [1, 2].

Diarrhoeal diseases have a detrimental impact on child growth and cognitive development [3]. Diarrhoeal diseases are associated with an increased risk of malnutrition in children [1]. Approximately 90% of diarrhoeal diseases occur in sub-Saharan and South Asian countries [4]. Although India has made considerable achievements in reducing infant and child mortality over the past 20 years, the episodes of preventable diseases like diarrhoea and pneumonia remain high. According to the National Family Health Survey (NFHS)-4, about 9% of under-five children had diarrhoea in the past 2 weeks preceding the survey during 2015–16 [5]. It is also notable that the prevalence of diarrhoea significantly varies across geographic regions of the country from as high as 13.1% in the central to as low as 4.2% in the northeast region [5]. In India, pneumonia and diarrhoeal diseases accounted for 50% of all under-five deaths [6]. The government of India has initiated several interventions to reduce the burden of diarrheal diseases. In 1975, the Integrated Child Development Scheme (ICDS) was launched to reduce the incidence of childhood malnutrition, morbidity, and mortality by providing supplementary nutrition and routine vaccination [7]. Besides, Water, Sanitation and Hygiene (WASH) trails, National Diarrhoeal Disease Control Program and policies for child health and nutritional programs have been initiated to control the incidence of diarrhoea-related morbidity and mortality in children.

Many socio-demographic, environmental, and behavioral factors are responsible for the occurrence of diarrhoeal disease among under-five children. Important socio-demographic factors include children’s age, place of residence, maternal education, and household economic condition [8–15]. Environmental factors such as drinking water, sanitation facilities, waste disposal, and dwelling characteristics are associated with childhood diarrhoea [11–13, 16, 17]. Furthermore, behavioral factors such as breastfeeding practices, eating habits, and handwashing practices are found to be significant factors of diarrhoea in children under five [12, 18, 19]. The Global Burden of Disease Study (2016) conducted in 195 countries yielded that child wasting, unsafe sanitation, low coverage of rehydration solution and unsafe water source are the leading risk factors of diarrhoeal disease in children under five worldwide [4]. A systematic review study in India indicated that diarrhoeal diseases in children occur due to a range of socio-economic factors including malnutrition, poor sanitation, and hygienic practices [15]. Studies also highlighted that breastfeeding practices, birth weight, and immunization coverage are significantly associated with childhood diarrhoeal diseases [15, 20, 21].

Despite high reported cases of diarrhoeal diseases, identifying the risk factors of childhood diarrhoea is relatively scant in India as compared to other developing countries. Moreover, malnutrition and early childhood mortality are unacceptably high in India. Prevention of diarrhoea is necessary to reduce the incidence of diarrhoea-related morbidity and mortality in children. In a context where the burden of diarrhoea-related mortality is high, it is imperative to investigate the factors associated with childhood diarrhoea. Therefore, this study aims to examine the socio-demographic and environmental factors associated with diarrhoeal disease in children under five in India using a nationally representative sample survey.

## Methods

### Study design

This study used secondary data collected from the fourth round of the National Family Health Survey (NFHS-4), conducted in 2015–16. A cross-sectional design was adopted in this study.

### Sample size and procedure

All living under-five children (*n* = 247,743) who participated in the NFHS-4 were enrolled in the study. The NFHS-4 is a nationally representative large-scale sample survey carried out across all the states and union territories of India. The samples were selected using a two-stage stratified sampling design. In the first stage, the clusters were selected using the method of probability proportional to size. In the second stage, complete household mapping and listing have been done in the selected clusters and 22 households were randomly selected from each cluster. The sampling frame used in this survey was the 2011 Indian Population and Housing Census. A detailed description of the sampling design and survey procedure is provided in the NFHS-4 national report [5].

### Outcome variable

The outcome variable of this study is diarrhoeal disease in children under five. Diarrhoea is defined as having three or more loose or watery stools in 24 h, as reported by the mother/caregiver in the past 2 weeks preceding the survey.

### Explanatory variables

Socio-demographic and environmental characteristics were included for explanatory variables in this study. The operational definition and coding of variables are included in Table 1. Socio-demographic characteristics include place of residence (rural and urban), caste (scheduled castes/scheduled tribes, other backward classes, and others), religion (Hindu, Muslim, and others), maternal education (illiterate/primary and secondary/above) father’s education (illiterate/primary and secondary/above), maternal age (15–24, 25–34, and 35–49 years), maternal body mass index [BMI] (underweight, normal, and overweight/obese), access to mass media (no and yes), household size (< 6 and 6+), wealth quintile (poorest, poorer, middle, richer, and richest), region (north, central, east, northeast, west, and south), age of the child (0–11, 12–23, 24–35, 36–47, and 48–59 months), sex of child (male and female), birth order (1, 1–3, and 4+) and birth weight (< 2.5 kg [low birth weight] and ≥ 2.5 kg [normal]).

**Table 1:** Description of explanatory variables included in the analysis, NFHS-4 (2015–16)

Variables	Operational definition and codes
**Socio-economic characteristics**	
Place of residence	Coded as 1 = Urban, 2 = Rural
Caste	Coded as 1 = Scheduled Castes, 2 = Scheduled Tribes, 3 = Other Backward Classes, 4 = Others
Religion	Coded as 1 = Hindu, 2 = Muslim, 3 = Others
Maternal education	Coded as 1 = Illiterate/Primary, 2 = Secondary, 3 = Higher
Father’s education	Coded as 1 = Illiterate/Primary, 2 = Secondary, 3 = Higher
Maternal age	Self-reported ages coded as 1 = 15–24, 2 = 25–34, 3 = 35–49 years
Maternal BMI	BMI was calculated from height and weight of the mother as per WHO standards: 1 = Underweight, 2 = Normal, 3 = Overweight/obese
Access to mass media	Measured from frequency of reading newspapers/magazines, listening radio and watching television: 1 = No, 2 = Yes
Household size	Classified as 1 = Less than 6, 2 = Above 6
Wealth quintile	Access to household assets including dwelling characteristics, categorized into 5 quintile: 1 = Poorest, 2 = Poorer, 3 = Middle, 4 = Richer, 5 = Richest
Region	Regions of residence as provided by NFHS: 1 = North, 2 = Central, 3 = East, 4 = Northeast, 5 = West, 6 = South
Age of child	Self-reported ages coded as 1 = 0–11, 2 = 12–23, 3 = 24–35, 4 = 36–47, 5 = 48–59 months
Sex of child	1 = Male, 2 = Female
Birth order	Categorized into 3 groups: 1 = 1, 2 = 1–3, 3 = 4+
Birth weight	Classified as 1 = < 2.5 kg (low birth weight), 2 = ≥2.5 kg (normal)
**Environmental characteristics**	
Availability of toilet facility	Coded as 1 = No facility, 2 = Facility
Sources of drinking water	Coded as 1 = Improved, 2 = Unimproved
Child stool disposal	Coded as 1 = Not safe, 2 = Safe
Main floor materials	Coded as 1 = Dirt, 2 = Non-dirt
Main roof materials	Coded as 1 = Thatch, 2 = Concrete/metal

Environmental characteristics include the availability of toilet facility, sources of drinking water, child stool disposal, and main floor and roof materials of the household. Household toilet facility was a dichotomous variable, classified as households having a toilet facility and households without a toilet facility. Any improved, unimproved, and shared latrine facility (flush or pit) were considered as households having a toilet facility, otherwise households using open defecation. Sources of drinking water were categorized as improved and unimproved sources. Improved sources of drinking water are protected from outside contamination, particularly from fecal matter [22]. Improved sources include piped water, public taps, standpipes, tube wells and boreholes, protected dug wells and springs, rainwater, and community reverse osmosis plants, while unimproved sources comprise unprotected dug well, unprotected spring, tanker/cart with a small tank, surface water, and bottled water. Child stool disposal was grouped into safe and unsafe disposal. Children’s stool was considered to be safely disposed, if a child used a toilet or latrine, or flushed into a toilet, or it was buried [5]. The main floor materials of the houses were categorized as dirt (e.g., mud/clay/earth, sand, and dung) and non-dirt (e.g., raw wood planks, palm, bamboo, brick, stone, parquet or polished wood, vinyl or asphalt, ceramic tiles, cement, carpet and polished stone/marble/granite). Likewise, the main roof materials of the dwelling unit were grouped into thatch (e.g., thatch/palm leaf, mud, plastic/polythene, rustic mat, palm/bamboo, timber, etc.) and metal/concrete (e.g., metal, wood, asbestos sheets, cement/concrete, tiles, slates, burnt bricks, etc.).

### Data analysis

Descriptive statistics were carried out to understand the distribution of socio-demographic and environmental characteristics for the study sample. The prevalence of diarrhoeal disease was estimated by selected explanatory variables. Pearson’s chi-square test was used and variables with *p* < 0.05 were included for further analysis. The sample weight was used for the estimation of percentage distribution. Bivariate and multivariate logistic regression models were employed to assess the socio-demographic and environmental factors associated with diarrhoea in children under five. The regression results are presented by unadjusted and adjusted odds ratio (OR) with 95% confidence interval (CI) and the results were considered to be statistically significant at *p* < 0.05. All the statistical analyses were performed using STATA version 14.0 (StataCorp LP, College Station, TX, USA).

## Results

### Socio-demographic characteristics

A total of 247,743 living children aged 0–59 months were included in the analysis. The majority of children who lived in rural areas (71.6%), affiliated to Hindu (78.6%), belonged to poor families (24.9% in the poorest wealth quintile and 21.8% in the poorer wealth quintile) and resided in the central and east regions of the country. About 43.5% of mothers had below the secondary level of education, while the corresponding figure for fathers was 31.6%. About 57.7% of mothers were in the age range of 25–34 years and one-fourth (24.8%) of them were underweight. Over one-third of mothers (34.3%) had no exposure to mass media. Respondents were almost equally distributed in all age groups. Among total children, 52.1% were male, 47.5% were in the birth order of 2–3, and 17.7% of children had low birth weight (Table 2).

**Table 2:** Socio-demographic characteristics of living children aged 0–59 years in India, NFHS-4 (2015–16)

Variables	Frequency (n)	Percentage (%)
Place of residence		
Urban	59,222	28.5
Rural	188,521	71.6
Caste		
Scheduled castes	46,486	22.4
Scheduled tribes	49,804	11.0
Other backward classes	97,011	46.1
Others	43,329	20.5
Religion		
Hindu	178,712	78.6
Muslim	39,004	16.6
Others	30,027	4.9
Maternal education		
Illiterate/primary	112,129	43.5
Secondary and above	135,614	56.5
Father’s education		
Illiterate/primary	13,864	31.6
Secondary and above	29,151	68.4
Maternal age in years		
15–24	80,714	34.9
25–34	142,212	56.7
35–49	24,817	8.4
Maternal BMI		
Underweight	57,793	24.8
Normal	152,834	60.4
Overweight/Obese	33,876	14.8
Access to mass media		
No	91,357	34.3
Yes	156,386	65.7
Household size		
< 6	104,964	43.3
6+	142,779	56.8
Wealth quintile		
Poorest	64,443	24.9
Poorer	58,294	21.8
Middle	49,588	19.9
Richer	41,472	18.4
Richest	33,946	15.1
Region		
North	46,775	13.2
Central	70,915	26.5
East	51,507	25.4
Northeast	35,761	3.5
West	17,706	12.9
South	25,079	18.4
Age of child in months		
0–11	48,295	19.3
12–23	49,284	20.0
24–35	49,084	19.8
36–47	51,497	20.9
48–59	49,583	20.0
Sex of child		
Male	128,609	52.1
Female	119,134	47.9
Birth order		
1	91,872	38.6
2–3	116,467	47.5
4+	39,404	13.9
Birth weight		
Low birth weight	31,928	17.7
Normal	155,254	82.4

### Environmental condition of the household

Around 46.5% of children living in the houses had no toilet facility. Only about 10% of children were drinking water from unimproved sources. About two-thirds (65.9%) of parents reported that their practice for child stool disposal was not safe. About 43.2% of children living in houses were made of dirt floor materials, and 14% of houses were made of thatch roof materials (Table 3).

**Table 3:** Environmental characteristics of living children aged 0–59 years in India, NFHS-4 (2015–16)

Environmental characteristics	Frequency (n)	Percentage (%)
Availability of toilet facility		
No facility	103,755	46.5
Facility	130,525	53.5
Sources of drinking water		
Unimproved	29,084	10.0
Improved	205,107	90.0
Child stool disposal		
Safe	83,545	34.1
Not safe	160,790	65.9
Main floor material		
Dirt	107,786	43.2
Non-dirt	126,668	56.9
Main roof material		
Thatch	32,407	14.0
Metal/concrete	193,533	86.0

### Prevalence of childhood diarrhoea by socio-demographic characteristics

The prevalence of diarrhoea was significantly higher in children living in rural areas as compared to those living in urban (9.6 vs. 8.2%) (*p* = 0.001). Childhood diarrhoea was common among scheduled caste (9.6%) and other backward class (9.6%) and Muslim children (9.9%). Maternal education was found to be significantly correlated with children’s diarrhoea occurrence (*p* < 0.001). However, father’s education was not significantly associated with child diarrhoea (*p* = 0.950). The occurrence of diarrhoea was substantially higher among those children whose mothers were in 15–24 years (10.7%), underweight (10.1%), and had no access to mass media (10.3%). Childhood diarrhoea was also higher in children from the poorest households (10.2%) and those from the central region of the country (13.1%). Furthermore, the prevalence of diarrhoeal disease was common among children aged 0–11 months (14.0%) and 12–23 months (13.4%), male children (9.5%), birth order above three (10.4%), and children with low birth weight (10.2%) (Table 4).

**Table 4:** Prevalence of diarrhoeal disease by socio-economic and demographic characteristics of respondents in India, NFHS-4 (2015–16)

Socio-economic and demographic characteristics	Prevalence (%)	*P*-value
Place of residence		0.001
Urban	8.2	
Rural	9.6	
Caste		< 0.001
Scheduled caste	9.6	
Scheduled tribe	8.1	
Other backward classes	9.6	
Others	8.8	
Religion		< 0.001
Hindu	9.2	
Muslim	9.9	
Others	7.3	
Maternal education		< 0.001
Illiterate/primary	9.6	
Secondary and above	8.8	
Father’s education		0.950
Illiterate/primary	9.6	
Secondary and above	9.2	
Maternal age in years		< 0.001
15–24	10.7	
25–34	8.5	
35–49	8.0	
Maternal BMI		< 0.001
Underweight	10.1	
Normal	9.2	
Overweight/Obese	8.1	
Access to mass media		< 0.001
No	10.3	
Yes	8.6	
Household size		0.519
< 6	9.08	
6+	9.28	
Wealth quintile		< 0.001
Poorest	10.2	
Poorer	9.5	
Middle	9.3	
Richer	8.5	
Richest	7.8	
Region		< 0.001
North	8.1	
Central	13.1	
East	8.7	
Northeast	4.2	
West	8.5	
South	6.5	
Age of child in months		< 0.001
0–11	14.0	
12–23	13.4	
24–35	8.5	
36–47	5.8	
48–59	4.6	
Sex of child		< 0.001
Male	9.5	
Female	8.9	
Birth order		< 0.001
1	8.7	
2–3	9.2	
4+	10.4	
Birth weight		< 0.001
Low birth weight	10.2	
Normal	8.6	

### Childhood diarrhoea by environmental condition

The prevalence of diarrhoea was found to be considerably higher in children of households who had no toilet facility compared to those whose households had a toilet facility (10.0 vs. 8.3%). The proportion of children with diarrhoea from households with improved drinking water sources was higher than those children whose households consumed unimproved sources (9.2 vs. 8.0%). The prevalence of diarrhoea was higher among households where child stool disposal practices were not safe (10.0%). The occurrence of diarrhoea was substantially higher in children living in houses having dirt floor (10.3%) and thatch roof materials (10.4%) (Table 5).

**Table 5:** Prevalence of diarrhoeal disease by environmental condition of respondents in India, NFHS-4 (2015–16)

Environmental characteristics	Prevalence (%)	*P-*value
Availability of toilet facility		< 0.001
No facility	10.0	
Facility	8.3	
Sources of drinking water		< 0.001
Unimproved	8.0	
Improved	9.2	
Child stool disposal		< 0.001
Safe	7.7	
Not safe	10.0	
Main floor material		< 0.001
Dirt	10.3	
Non-dirt	8.2	
Main roof material		< 0.001
Thatch	10.4	
Metal/concrete	8.8	

### Socio-demographic factors associated with diarrhoea among under-five children

In bivariate analysis, it is found that all the selected socio-demographic factors were significantly associated with children’s diarrhoea in the past 2 weeks. Multivariate analysis of this study revealed that children living in rural areas had 5% higher likelihood of diarrhoeal disease (aOR: 1.05; 95% CI: 1.01, 1.09) as compared to living in urban. Children belonged to the scheduled tribe (aOR: 0.83; 95% CI: 0.79, 0.89) and other castes (aOR: 0.92; 95% CI: 0.88, 0.97) were less likely to develop diarrhoea as compared to scheduled caste children. The likelihood of childhood diarrhoea was 18% higher among Muslim children (aOR: 1.18; 95% CI: 1.13, 1.24) as compared to children from Hindu religion. Participants whose mothers had the secondary and above level of education were associated with 9% decreased odds of diarrhoea (cOR: 0.92; 95% CI: 0.88, 0.97) as compared to those mothers who had no formal education or had primary level of education in crude analysis. However, this association was not significant in the adjusted analysis.

Unadjusted analysis of this study also found that the likelihood of childhood diarrhoea decreased with an increase in maternal age. Children of mothers aged 25–34 years (cOR: 0.77; 95% CI: 0.75, 0.80) and 35–49 years (cOR: 0.73; 95% CI: 0.69, 0.77) were less likely to suffer from the diarrhoeal disease than those children of mothers aged 15–24 years. Maternal BMI also had a significant association with children’s diarrhoea occurrence in unadjusted analysis. Children of underweight women (cOR: 1.11; 95% CI: 1.08, 1.15) were 11% more likely and children of overweight/obese women (cOR: 0.87; 95% CI: 0.83, 0.91) were l3% less likely to develop diarrhoea as compared to children of mothers who had normal BMI. The results also revealed that access to mass media of women was found to be a protective factor against childhood diarrhoea. Children of women who had mass media exposure were associated with 18% decreased odds of diarrhoea (cOR: 0.82; 95% CI: 0.80, 0.95) than those women who were not exposed to mass media. Wealth status of the household was found to have a strong association with childhood diarrhoea. Children in the richest wealth quintile were 26% less likely to have diarrhoea as compared to those children in the poorest wealth quintile (cOR: 0.74; 95% CI: 0.71, 0.78). The occurrence of diarrhoea was also varied across geographical regions. After adjusting for socio-demographic and environmental characteristics, children from the central (aOR: 1.61; 95% CI: 1.52, 1.70) and west (aOR: 1.08; 95% CI: 1.01, 1.15) regions were more likely and children from the northeast (aOR: 0.49, 95% CI: 0.43, 0.56) and south (aOR: 0.80, 95% CI: 0.75, 0.85) regions were less likely to experience diarrhoea as compared to children from the north region of the country.

The risk of diarrhoea was decreased by 4–71% in children aged 12–23 months (aOR: 0.96; 95% CI: 0.92, 1.00), 24–35 months (aOR: 0.58; 95% CI: 0.55, 0.61), 36–47 months (aOR: 0.39; 95% CI: 0.37, 0.41) and 48–59 months (aOR: 0.29; 95% CI: 0.28, 0.31) as compared to those children aged 0–11 months. Female children were associated 8% decreased odds of diarrhoea (aOR: 0.92; 95% CI: 0.89, 0.95) than male children. Birth order was found to be positively related to diarrhoeal disease in children. The odds of diarrhoeal disease increased by 22% in fourth or higher birth order children (cOR: 1.22; 95% CI: 1.17, 1.27) as compared to first birth order children. Low birth weight also increased the risk of diarrhoea by 19% (cOR: 1.19; 95% CI: 1.15, 1.24) as compared to children with normal birth weight (Table 6).

**Table 6:** Bivariate and multivariate logistic regression analysis of socio-demographic and environmental factors associated with childhood diarrhoea in India, NFHS-4 (2015–16)

Characteristics	Crude OR	95% CI		Adjusted OR	95% CI	
**Socio-demographic**						
Place of residence						
Urban	1.00			1.00		
Rural	1.18*	1.14	1.22	1.05*	1.01	1.09
Caste						
Scheduled caste	1.00			1.00		
Scheduled tribe	0.83*	0.78	0.87	0.83*	0.79	0.89
Other backward classes	1.00	0.96	1.04	0.97	0.93	1.01
Others	0.91*	0.87	0.95	0.92*	0.88	0.97
Religion						
Hindu	1.00			1.00		
Muslim	1.09*	1.05	1.13	1.18*	1.13	1.24
Others	0.79*	0.73	0.85	1.07	0.99	1.16
Maternal education						
Illiterate/primary	1.00			1.00		
Secondary and above	0.91*	0.88	0.93	1.01	0.98	1.05
Maternal age in years						
15–24	1.00			–	–	–
25–34	0.77*	0.75	0.80	–	–	–
35–49	0.73*	0.69	0.77	–	–	–
Maternal BMI						
Underweight	1.11*	1.08	1.15	–	–	–
Normal	1.00			–	–	–
Overweight/Obese	0.87*	0.83	0.91	–	–	–
Access to mass media						
No	1.00			1.00		
Yes	0.82*	0.80	0.85	0.98	0.94	1.02
Wealth quintile						
Poorest	1.00					
Poorer	0.92*	0.88	0.96	–	–	–
Middle	0.90*	0.86	0.93	–	–	–
Richer	0.82*	0.78	0.85	–	–	–
Richest	0.74*	0.71	0.78	–	–	–
Region						
North	1.00			1.00		
Central	1.71*	1.63	1.79	1.61*	1.52	1.70
East	1.09*	1.03	1.14	1.02	0.97	1.08
Northeast	0.49*	0.44	0.55	0.49*	0.43	0.56
West	1.05	0.99	1.11	1.08*	1.01	1.15
South	0.79*	0.75	0.83	0.80*	0.75	0.85
Age of child in months						
0–11	1.00			1.00		
12–23	0.95*	0.92	0.99	0.96*	0.92	1.00
24–35	0.57*	0.55	0.60	0.58*	0.55	0.61
36–47	0.38*	0.36	0.40	0.39*	0.37	0.41
48–59	0.30*	0.28	0.31	0.29*	0.28	0.31
Sex of child						
Male	1.00			1.00		
Female	0.93*	0.91	0.96	0.92*	0.89	0.95
Birth order						
1	1.00			1.00		
2–3	1.07*	1.03	1.10	1.01	0.98	1.05
4+	1.22*	1.17	1.27	1.02	0.97	1.07
Birth weight						
Low birth weight	1.19*	1.15	1.24	–	–	–
Normal	1.00			–	–	–
**Environmental**						
Availability of toilet facility						
No facility	1.00			1.00		
Facility	0.82*	0.80	0.84	0.97	0.94	1.02
Sources of drinking water						
Unimproved	1.00			1.00		
Improved	1.17*	1.12	1.24	1.03	0.98	1.09
Child stool disposal						
Safe	1.00			1.00		
Not safe	1.32*	1.28	1.36	1.06*	1.02	1.11
Main floor material						
Dirt	1.28*	1.25	1.32	1.08*	1.03	1.12
Non-dirt	1.00			1.00		
Main roof material						
Thatch	1.21*	1.16	1.25	1.08*	1.04	1.13
Metal/concrete	1.00			1.00		

* *p* < 0.05; *OR* Odds ratio, *CI* Confidence interval

### Environmental factors associated with diarrhoea among under-five children

Crude analysis demonstrated that children having a toilet facility in their houses had 18% less likely to have diarrhoeal diseases (cOR: 0.82; 95% CI: 0.80, 0.84) than those children who had no toilet facility in their houses. Surprisingly, children living in households having improved sources of drinking water were associated with higher odds of diarrhoea (cOR: 1.17; 95% CI: 1.12, 1.24) compared to those households having unimproved sources of drinking water. Child unsafe stool disposal was associated with an elevated risk of diarrhoea (aOR: 1.06; 95% CI: 1.02, 1.11) as compared to safe disposal of child stool. Children from households having dirt floor materials were associated with 8% higher likelihood of diarrhoea (aOR: 1.08; 95% CI: 1.03, 1.12) compared to those whose households had non-dirt floor materials. Similarly, children living in houses having thatch roof materials were 8% more likely to experience diarrhoeal disease (aOR: 1.08; 95% CI: 1.04, 1.13) than those children from houses with metal or concrete roof materials (Table 6).

## Discussion

The present study has examined the socio-demographic and environmental factors associated with diarrhoea in children younger than 5 years in India. Although the prevalence of childhood diarrhoea has been reduced over the recent period, the burden of this preventable disease remains high in India. As per the NFHS-4, approximately 9% of under-five children suffered from diarrhoeal diseases [5].

The findings of this study revealed that children living in rural areas were more likely to experience diarrhoea than those living in urban areas. This finding is in line with previous studies conducted in India [13] and Jamma district of Ethiopia [17]. This could be due to limited access to healthcare and sanitation facilities in rural areas [17]. Caste and religion were significantly associated with childhood diarrhoeal disease. The present study found that children from scheduled tribes and other caste groups had a lower risk of diarrhoea compared with those from scheduled caste children. This finding is consistent with a study carried out in India [13]. Moreover, Muslim children were 18% more likely to develop the diarrhoeal disease as compared to Hindu children. This might be due to scheduled caste and Muslim children have lower access to improved sources of drinking water and sanitation facilities compared to other socio-religious groups of children.

Concerning maternal characteristics, maternal education, and access to mass media were included in the multivariate analysis and none of these variables were found to be significantly associated with diarrhoea in children. However, the crude analysis of this study showed that all maternal factors were significantly determined diarrhoea in children. For instance, women’s secondary or higher level of education was associated with 9% reduced likelihood of diarrhoea in children as compared to those who had no education or primary level of education. A similar finding is also reported in other studies conducted in Bangladesh [9] and different parts of Ethiopia [8, 11, 14]. This could be explained by hygiene practices, child feeding and caring practices, and improved living conditions of an educated mother [14]. Furthermore, this study also found that underweight mothers were associated with 11% increased risk of diarrhoea in children. Children born to underweight women may also become malnourished and have a weak immune system. Therefore, malnourished children are highly susceptible to infectious diseases including diarrhoea. Mass media exposure of mothers was found to be a protective factor of childhood diarrhoea. In unadjusted analysis, the study found that children whose mothers had mass media access were less likely to have diarrhoea disease.

The crude analysis of this study also indicated that wealth status of the household was significantly associated with children’s recent diarrhoea occurrence. The findings of the present study revealed that the likelihood of diarrhoea was reduced by 26% in children from the richest wealth quintile as compared to those from the poorest wealth quintile. A similar finding is also found in earlier studies conducted in India [13] and other developing countries [9, 23]. Wealth quintile variable was excluded from the multivariate analysis since household wealth was measured from a range of consumer items, sources of drinking water, and sanitation facilities including dwelling characteristics. Many of these characteristics were included in the multivariate analysis as environmental factors.

Geographical region of residence also made significant variations in the prevalence of diarrhoeal diseases. The present study indicated that children from the central region were 61% and children resided in the western region were 8% more likely to experience diarrhoea as compared to children from the south region. In contrast, children from the northeast region were 51% and children from the south region were 20% less likely to develop diarrhoea. Geographical differences in diarrhoeal disease have also been reported in a study conducted in India [24]. This could be due to unequal access to healthcare and inequity in the provisioning of drinking water and sanitation facilities [25].

This study found that the risk of diarrhoea was decreased by 43–70% in children aged 24 to 59 months as compared to infants (children aged 0–11 months). An earlier study conducted in Kashmir (India) indicated maximum reported cases of diarrhoeal disease between the ages of 6 to 11 months [26]. Likewise, a study carried out in Jamma district of Ethiopia found that the likelihood of developing diarrhoea was more than twice among children aged 6 to 23 months as compared to children aged 2 years or above [17]. A similar finding is also found in a study done in Bangladesh [9]. This is because, in the infant age, children are exposed to different contaminated agents leading to infectious diseases while crawling and walking [14]. Female children were found to have 8% reduced risk of diarrhoea than male children. A study conducted in Bangladesh also revealed that male children had higher odds of diarrhoea than female children [9]. A similar finding is also reported in a study carried out in rural parts of western Maharashtra [27]. The current study further demonstrated that children with low birth weight were more likely to have diarrhoea as compared to those children with normal birth weight. A rural community-based study in south India indicated that low birth weight in children increased the risk of diarrhoeal disease by almost three-folds [21].

Among environmental factors, child stool disposal, floor and roof materials of the household unit were significantly associated with diarrhoea in under-five children. In agreement with previous research [11], the current study also revealed that unsafe disposal of child stool was associated with 6% increased likelihood of diarrhoea as compared to safe child stool disposal. This might be due to disposed child stool contaminated the water storage that may cause diarrhoeal diseases. The odds of having diarrhoea were 8% higher among children living in houses with dirt floor materials as compared to those living in houses with non-dirt floor materials. This finding is consistent with several studies conducted in Ethiopia [8, 10, 11, 17]. Similarly, the risk of developing the diarrhoeal disease was 8% higher in children from households having thatch roof materials than those from households having metal or concrete roof materials. This finding is also in line with studies conducted in Ethiopia [16]. This could be because the dirty floor and thatch roof materials of the dwelling cause the transmission of pathogens, which may increase the risk of diarrhoeal diseases [8].

### Limitations and strengths

The current study findings should be discussed in light of some limitations. First, causality cannot be assumed from this analysis due to the cross-sectional nature of data. Further research is needed using longitudinal data to examine the potential pathways for the occurrence of diarrhoea in children. Second, this study used self-reported retrospective information. Therefore, potential recall bias might have been introduced in this study [28]. Lastly, since the study used secondary data, it was not possible to include all important potential factors of diarrhoeal disease, particularly behavioral factors in the analysis due to the paucity of information in the dataset.

Besides the above limitations, this study provides comprehensive evidence on the socio-demographic and environmental factors associated with diarrhoeal disease in children under five using a large-scale demographic and health survey in India. The study used a large number of samples with nation-wide representation. Further, this study provides more evidence on factors that need to be targeted by public health interventions in an effort to further reduce the prevalence of diarrhoea in children.

## Conclusions

A number of socio-demographic such as rural-urban residence, caste, religion, region, child’s age and sex are associated with the risk of diarrhoea among under-five children in India. Diarrhoea is common among rural, scheduled caste, Muslim and poor families’ children. Concerning environmental factors, stool disposal practices in the household, main floor and roof materials of the household unit are risk factors for diarrhoeal disease. Interventions should be made in improving MCH to reduce the burden of diarrhoea and diarrhoea-related mortalities in children under five. Targeted approach should be initiated to mitigate the problem of the poor health status of children by providing adequate health care among socio-economically disadvantaged women and children. The policy-makers and stakeholders should address adverse environmental conditions by the provision of latrine and improved housing facilities.

## Data Availability

The dataset analysed during the current study are available in the Demographic and Health Surveys (DHS) repository, https://dhsprogram.com/data/available-datasets.cfm.

## Abbreviations

BMI: Body mass index
CI: Confidence interval
ICDS: Integrated Child Development Scheme
MCH: Maternal and child health
NFHS: National Family Health Survey
OR: Odds ratio
WHO: World Health Organization
